# An Overview of Meta-Analyses on the Surgical Stabilization of Rib Fractures in Adults: A Narrative Umbrella Review (2020–2025)

**DOI:** 10.3390/jcm15103648

**Published:** 2026-05-09

**Authors:** Maria Chiara Sibilia, Francesca Romboni, Sara Franzi, Lorenzo Bramati, Maria Carmela Andrisani, Mario Nosotti, Davide Tosi

**Affiliations:** 1 Department of Pathophysiology and Transplantation, University of Milan, 20122 Milan, Italy; 2 Department of Cardio-Thoraco-Vascular Diseases, Foundation IRCCS Ca’ Granda—Ospedale Maggiore Policlinico, 20122 Milan, Italy; 3 Department of Cardio-Thoraco-Vascular Diseases, ASST Brianza—Ospedale di Vimercate, 20871 Vimercate, Italy; 4 Radiology Department, Fondazione IRCCS Ca’ Granda Ospedale Maggiore Policlinico, 20122 Milan, Italy; 5 Department of Surgical Sciences and Integrated Diagnostics (DISC), School of Medical and Pharmaceutical Sciences, University of Genoa, 16132 Genoa, Italy

**Keywords:** meta-analyses, rib fractures, fracture management, surgical stabilization, flail chest, non-operative management, patients’ outcome

## Abstract

**Background:** Rib fractures are a common cause of morbidity in trauma patients. The surgical stabilization of rib fractures (SSRF) has gained increasing attention as a therapeutic option; however, evidence from multiple meta-analyses remains heterogeneous. **Methods:** We performed an overview of 11 meta-analyses, including a total of 1,117,849 adult patients (narrative umbrella review), published between November 2020 and November 2025 to summarize and critically appraise high-level evidence comparing SSRF with non-operative management (NOM) in adults with traumatic rib fractures. PubMed (MEDLINE) and Embase were searched for eligible meta-analyses. Outcomes of interest included mechanical ventilation duration, pneumonia, ICU and hospital length of stay, mortality, pain, quality of life, and need for tracheostomy. **Results:** Eleven meta-analyses met the inclusion criteria. Across outcomes, the direction of effect generally favored SSRF in selected patients, particularly with respect to a shorter duration of mechanical ventilation (mean difference up to approximately 4–6 days), reduced pulmonary complications (risk ratio approximately 0.4–0.7), shorter ICU and hospital stay, and improved pain control. However, results varied substantially across studies. A consistent mortality benefit was not observed. Subgroup analyses suggested that the benefits of SSRF were more pronounced in patients with flail chest, severe fracture patterns, and early surgery, whereas findings were less consistent in elderly patients and in patients with less severe injuries. **Conclusions:** This narrative umbrella review suggests that SSRF is associated with improved short-term outcomes in selected adult patients with traumatic rib fractures but should not be considered a universal standard of care. Careful patient selection, timing of intervention, and multidisciplinary evaluation remain essential. Further high-quality prospective studies are needed to better define optimal indications and management strategies.

## 1. Introduction

Thoracic injury is a common and potentially severe component of acute trauma. Rib fractures represent the most frequent form of thoracic injury, ranging from simple isolated fractures to complex patterns associated with chest wall instability and flail chest [1]. Multiple rib fractures and flail chest are associated with high mortality (approximately 19%), mainly due to respiratory complications, such as pneumonia, respiratory distress, and hemorrhage. Chest trauma represents approximately 15% of trauma admissions, and almost 60% results from car accidents [2]. Rib fractures occur in approximately 10% of patients with blunt chest trauma and in up to 50% of polytrauma cases, contributing significantly to trauma-related morbidity and mortality. Younger patients are more often involved in car accidents, whereas, in the elderly, injuries related to decreased bone density, such as ground-level falls, are more common [2,3].

Rib fractures can be classified according to fracture morphology (simple, wedge, or complex) and displacement, most commonly involving the fourth to tenth ribs in the anterolateral region [4].

Clinically, they are often associated with severe pain due to intercostal nerve involvement, which may impair respiratory mechanics, limit effective coughing, and predispose patients to pulmonary complications such as atelectasis and pneumonia. Even in the absence of radiological flail chest, patients with multiple rib fractures exhibit a substantially increased risk of respiratory failure, prolonged mechanical ventilation, and intensive care unit (ICU) admission [4].

**Figure 1:**
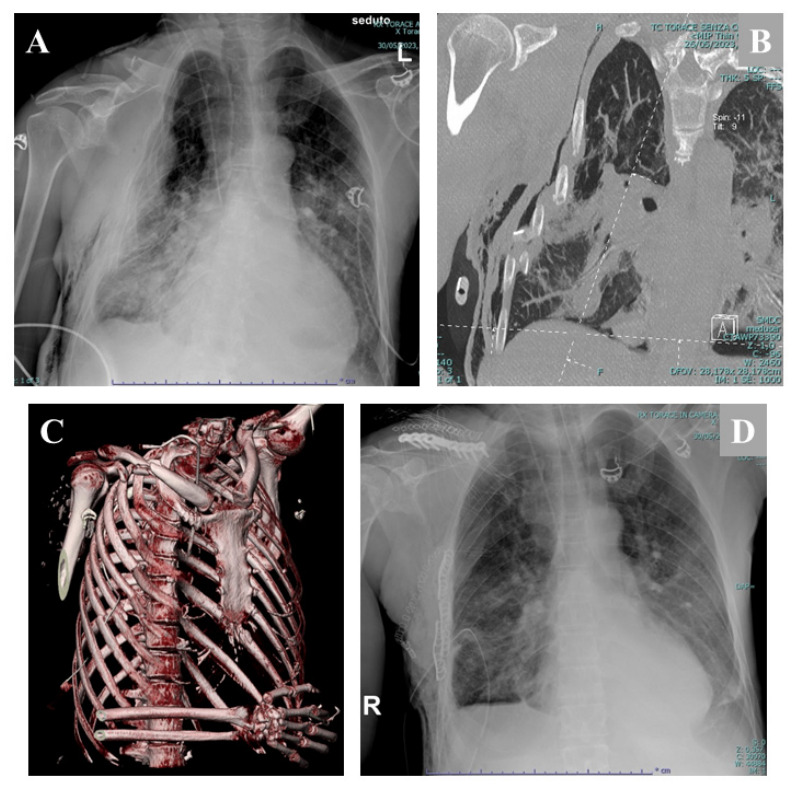
(**A**) Chest X-ray (AP projection): Multiple displaced rib fractures involving the right hemithorax are observed, with areas of pulmonary parenchymal contusion. A right-sided pneumothorax is present, with associated subcutaneous emphysema of the right chest wall. A displaced fracture of the mid-shaft of the right clavicle is also noted. (**B**) Chest CT (2D Coronal MIP reconstruction): In the same patient, the CT image demonstrates intrathoracic displacement of multiple right rib fracture fragments. Extensive subcutaneous emphysema is visible along the right chest wall. (**C**) Chest CT (3D reconstruction): The 3D CT image clearly demonstrates complex bifocal rib fractures involving the third to seventh ribs in the anterolateral region of the right hemicostat. (**D**) Postoperative chest X-ray (AP projection): Status post-surgical stabilization of right rib fractures with locking rib plates. The rib fracture fragments appear well aligned. There is near-complete resolution of the previously noted pulmonary contusion and of the subcutaneous emphysema of the chest wall.

Flail chest, defined as paradoxical movement of a chest wall segment resulting from fractures of at least three contiguous ribs in two or more locations, represents the most severe manifestation of rib fracture injury (Figure 1). 

This condition is frequently associated with high-energy trauma and concomitant injuries and is linked to significant respiratory compromise, prolonged ICU length of stay (ICU-LOS), and increased mortality [5].

Historically, rib fractures were managed almost exclusively with non-operative strategies, including analgesia and respiratory support. Over the past two decades, however, the surgical stabilization of rib fractures (SSRF) has emerged as a therapeutic option for selected patients, particularly those with flail chest, severe displacement, or respiratory compromise. Multiple randomized trials, observational studies, and meta-analyses have evaluated the impact of SSRF on clinically relevant outcomes, including mechanical ventilation duration, pulmonary complications, ICU-LOS and hospital stay, mortality, and pain control.

Consequently, the available literature has rapidly expanded, resulting in a growing number of meta-analyses addressing similar clinical questions but reporting partially heterogeneous results, often influenced by differences in patient selection, injury patterns, surgical timing, and study design. While individual meta-analyses provide valuable information, clinicians may face difficulties in interpreting the overall body of evidence and in identifying areas of consensus and uncertainty.

Therefore, we performed a narrative umbrella review (an overview of meta-analyses) of the meta-analyses published between November 2020 and November 2025 to provide a structured synthesis of high-level evidence comparing SSRF with non-operative management in adults with traumatic rib fractures. Our aim was to summarize consistent findings, explore sources of heterogeneity, and identify remaining evidence gaps relevant to clinical practice.

## 2. Materials and Methods

Study design: This study was designed as a narrative umbrella review (an overview of meta-analyses) aimed at summarizing and critically synthesizing the highest level of available evidence on the management of traumatic rib fractures in adults. The objective was not to perform a new quantitative meta-analysis, but rather to provide a structured qualitative synthesis of published meta-analyses comparing the surgical stabilization of rib fractures (SSRF) with non-operative management (NOM), highlighting areas of agreement, heterogeneity, and evidence gaps.

Search strategy: Two independent authors conducted a systematic literature search of the PubMed (MEDLINE) and Embase databases to identify relevant meta-analyses published between November 2020 and November 2025. The search strategy combined the following keywords and Medical Subject Headings (MeSH), adapted as appropriate for each database: “rib fractures” AND “surgical stabilization” OR “surgical management of rib fractures”. Reference lists of eligible articles were also screened to identify additional relevant publications.

Eligibility criteria: Studies were eligible for inclusion if they met the following criteria:

- Meta-analyses or systematic reviews with quantitative synthesis (meta-analysis);
- Adult population (≥18 years);
- Comparison between SSRF and NOM;
- Reporting at least one clinically relevant outcome related to respiratory function, complications, length of stay, mortality, pain, or quality of life;
- Published in English and indexed in MEDLINE or Embase.

Exclusion criteria were

- Studies including pediatric populations;
- Narrative reviews without meta-analytic synthesis;
- Meta-analyses focusing on non-traumatic rib fractures or non-surgical interventions only;
- Studies not available as full text.

**Figure 2:**
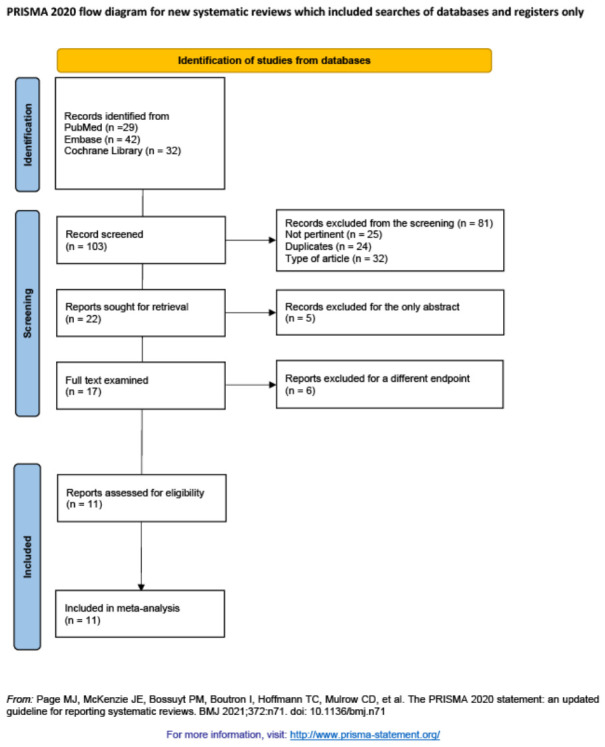
Flowchart outlining the process for selecting the meta-analyses we included in the study [6].

Study selection: After removing the duplicates, titles and abstracts were independently screened by two authors. Full-text articles were then assessed for eligibility. Disagreements were resolved by consensus. The study selection process is summarized in a flowchart (Figure 2).

Data extraction and outcomes: From each included meta-analysis, the following data were extracted—first author, year of publication, number of included patients, comparison groups, and reported outcomes.

Outcomes of interest included

- Duration of mechanical ventilation;
- Incidence of pneumonia;
- Length of stay in intensive care unit;
- Length of stay in hospital;
- Mortality;
- Need for tracheostomy;
- Pain and quality of life, when available.

Data synthesis and methodological considerations: Given the nature of an umbrella review, no additional pooling of effect sizes across meta-analyses was performed. Results were synthesized in a structured qualitative manner, focusing on the direction and consistency of reported effects.

A potential overlap of primary studies across included meta-analyses was acknowledged and considered in the interpretation of findings. Therefore, results are presented descriptively rather than as cumulative quantitative estimates.

Even if the primary aim of this review was to provide an overview of recent high-level evidence and its clinical implications rather than a methodological ranking of included meta-analyses, we performed a formal assessment of methodological quality (e.g., AMSTAR-2), as reported in Supplementary Table S1. Overall, the methodological quality of the included systematic reviews, as assessed using the AMSTAR-2, ranged from moderate to low. While several key domains were adequately addressed, some recurring limitations were observed, particularly the lack of a comprehensive list of excluded studies, an incomplete consideration of the risk of bias in the interpretation of findings, and an only partial assessment of publication bias. Protocol registration was inconsistently reported across all reviews. These methodological limitations may affect the overall confidence in the evidence and should be taken into account when interpreting the results.

Ethics: Ethical approval was not required, as this study was based exclusively on published data and did not involve human participants.

## 3. Results

A total of 11 meta-analyses meeting the inclusion criteria were included. These meta-analyses compared SSRF with non-operative management in adult patients with traumatic rib fractures and reported one or more of the outcomes of interest (Table 1A,B). Across included meta-analyses, results were synthesized descriptively, focusing on the direction and consistency of effects. Because meta-analyses differed substantially in size, included study designs, and populations, the frequency of findings favoring SSRF should be interpreted as a qualitative indicator rather than a quantitative weighting.

Duration of mechanical ventilation (MV): Most included meta-analyses (9 out of 11) reported a shorter duration of mechanical ventilation in the SSRF group compared with NOM, although the magnitude of effect varied across studies and patient subgroups. Two meta-analyses did not find a statistically significant difference between groups, as highlighted with an asterisk (*) in Table 1A [3,7]. Subgroup findings suggested that the potential benefit may be more consistent in flail chest and when SSRF is performed early (≤72 h), whereas results in older patients were less consistent [2].

Pneumonia: The majority of meta-analyses reported a lower incidence of pneumonia after SSRF than after NOM. However, subgroup analyses in some reports suggested that the effect may be attenuated in older patients and may differ according to fracture pattern (flail vs. non-flail) and baseline respiratory status.

ICU length of stay: Overall, most included meta-analyses (8 out of 11) reported a shorter ICU length of stay in patients undergoing SSRF, although heterogeneity was substantial. In Zhao [3], findings differed by subgroup, with potential benefit in flail chest but less consistent results in older patients.

Hospital length of stay: Findings for hospital length of stay were less consistent than for ICU stay and mechanical ventilation. Some meta-analyses (4 out of 11) reported significantly shorter hospital stay after SSRF, evident as negative values of mean difference (MD): Long et al., *p* = 0.001; Wijffells et al., *p* = 0.01; Sawyer et al., *p* = 0.03; He et al., *p* = 0.00001, whereas others found no difference or results favoring NOM in selected subgroups, particularly among older patients [12].

Mortality: A consistent mortality reduction with SSRF was not observed across all included meta-analyses. While some meta-analyses suggested a potential reduction in mortality, many reported no statistically significant difference, likely reflecting heterogeneity in populations, injury severity, and study design.

Pain and quality of life: Pain and QoL outcomes were inconsistently reported across meta-analyses. Where available, SSRF was generally associated with improved pain scores and/or reduced narcotic consumption. For example, in Long et al., the numeric pain score (NPS) was significantly lower after surgery than in the conservative group. Similarly, Apampa et al. reported a marked improvement in cough and deep breathing-induced pain at 1 week after surgery, and He reported a lower pain score in patients who underwent surgical fixation [9,10,14]. Nonetheless, definitions, follow-up intervals, and measurement tools varied substantially.

Tracheostomy: Evidence regarding tracheostomy rates was mixed. Some meta-analyses reported lower tracheostomy rates with SSRF, whereas others reported no significant differences. The inconsistency in results may be explained by heterogeneity across studies, including differences in ventilatory management strategies, the timing of both tracheostomy and surgical stabilization, variability in patient selection and injury severity, and relatively low event rates, which together may limit statistical power and contribute to divergent findings.

## 4. Discussion

As an overview of meta-analyses, the present work provides a high-level synthesis of recent evidence comparing the surgical stabilization of rib fractures (SSRF) with non-operative management (NOM) in adult patients with traumatic rib fractures. Overall, the direction of effect across most clinically relevant outcomes tends to favor SSRF in selected patients, particularly regarding mechanical ventilation duration, pulmonary complications, ICU and hospital length of stay, and pain control [1,17]. However, these findings should be interpreted in light of substantial clinical and methodological heterogeneity across the included meta-analyses.

One of the most relevant observations emerging from this umbrella review is that the apparent benefit of SSRF is not uniform across all patient populations. Heterogeneity across meta-analyses likely reflects differences in patient selection, fracture patterns, baseline respiratory status, and the timing of surgical intervention. In particular, patients with flail chest, severe displacement, or respiratory compromise appear to derive the most consistent benefit from SSRF, whereas the inclusion of less severe fracture patterns may attenuate treatment effects.

Surgical timing represents another key determinant of outcome. Several meta-analyses and underlying studies suggest that early stabilization, generally within 48–72 h after injury, is associated with improved respiratory outcomes, shorter ICU and hospital stay, and reduced complications. Additionally, a recent randomized controlled trial demonstrated that SSRF within 48 h significantly decreased levels of inflammatory cytokines and infection biomarkers, resulting in a general reduction in hospital costs [18]. Conversely, delayed intervention may reduce the magnitude of benefit, although selected patients may still experience clinical improvement when early surgery is not feasible.

Age also appears to play a significant role in modulating treatment effects. While SSRF may offer advantages in younger or physiologically robust patients, evidence in elderly populations is less consistent. Some meta-analyses and position statements suggest that, in older patients, the magnitude of benefit in terms of mechanical ventilation duration, pulmonary complications, and length of stay is attenuated compared with younger cohorts, with pooled estimates often showing smaller effect sizes and, in some cases, no statistically significant differences versus non-operative management [19]. This finding may reflect a combination of factors, including reduced physiological reserve, a higher burden of comorbidities, and increased susceptibility to perioperative complications, as well as differences in treatment goals and thresholds for escalating care. Importantly, the available evidence is largely derived from observational studies with heterogeneous age cut-offs and limited stratified analyses, which further complicates interpretation. Therefore, rather than indicating a lack of efficacy, these findings underscore the need for careful patient selection and multidisciplinary decision-making in elderly patients, integrating frailty, baseline functional status, and overall risk–benefit assessment. Some meta-analyses suggest that older patients may achieve outcomes comparable with optimized non-operative management, highlighting the importance of careful patient selection and multidisciplinary evaluation in this subgroup [19].

Importantly, the current evidence base is largely derived from retrospective studies, which are inherently susceptible to selection, indication, and survivor biases. These limitations are further amplified in meta-analyses that pool heterogeneous observational data, often with variable outcome definitions, an inconsistent reporting of surgical techniques, and limited long-term follow-up. The overlap of primary studies across meta-analyses further complicates interpretation and precludes quantitative aggregation at the umbrella level.

From a technical and perioperative perspective, a wide variety of surgical approaches and fixation systems are currently employed in clinical practice. However, no high-quality evidence demonstrates the superiority of one fixation method over another, and technical considerations remain largely dependent on surgeon experience and institutional resources. Similarly, perioperative pain management strategies vary substantially across centers, and standardized protocols integrating surgical stabilization with optimized analgesia may play a critical role in maximizing clinical benefit. Traditional techniques include intravenous opioid analgesia, thoracic epidural anesthesia (TEA), and paravertebral blocks (PVBs), although these may be contraindicated in some cases. More recently, novel techniques such as fascial plane blocks (erector spinae, serratus anterior), thoracoscopic intercostal blocks, and intercostal nerve cryoablation have shown effective analgesia with fewer side effects; erector spinae plane block (ESPB), for example, appears promising for its safety profile and sustained pain relief. Establishing standardized perioperative pain management protocols would help to optimize patient selection for surgery and improve postoperative outcomes [20].

Taken together, these findings support the view that SSRF should not be regarded as a universal standard of care, but rather as a valuable therapeutic option within a tailored, patient-focused treatment strategy. The integration of patient characteristics, injury severity, respiratory status, and institutional expertise is essential to optimize outcomes.

Future research should focus on well-designed prospective studies and randomized controlled trials with standardized inclusion criteria, uniform outcome definitions, and longer follow-up, with particular attention to elderly patients, non-flail fracture patterns, and the optimal timing of intervention. Such efforts are necessary to refine clinical indications, reduce practice variability, and strengthen the evidence base guiding rib fracture management.

## 5. Conclusions

Rib fractures represent a frequent and clinically relevant condition in polytrauma patients and are associated with substantial morbidity and healthcare resource utilization.

This narrative umbrella review of recent meta-analyses suggests that the surgical stabilization of rib fractures (SSRF), when compared with non-operative management, is associated with improved short-term clinical outcomes in selected adult patients, including reductions in the duration of mechanical ventilation, the incidence of pneumonia, the length of ICU and hospital stay, and improved pain control [21]. However, no consistent mortality benefit emerges across all patient populations.

Importantly, the observed benefits of SSRF appear to be strongly influenced by patient selection, injury pattern (flail versus non-flail chest), age, and the timing of surgical intervention, with early stabilization showing the most consistent association with favorable outcomes. Conversely, in elderly patients and in less severe fracture patterns, non-operative management may achieve comparable results.

The current body of evidence is largely derived from retrospective studies and heterogeneous meta-analyses, with variability in indications, surgical techniques, perioperative management, and outcome definitions. As a consequence, SSRF should not be considered a universal standard of care, but rather a valuable therapeutic option within a multidisciplinary, individualized treatment strategy.

Further high-quality prospective studies and randomized controlled trials are needed to better define optimal indications, timing, and perioperative protocols for SSRF, with the aim of reducing treatment variability and improving patient-focused outcomes.

From a clinical perspective, this overview may assist trauma teams in integrating current evidence into patient-specific decision-making for rib fracture management.

## Figures and Tables

**Table 1:** (**A**) List of parameters assessed in the included studies, comparing surgical fixation of rib fractures (SSRF) with the non-operative (NOM) approach. (**B**) The quality of life (QoL) was assessed as pain score or as duration of narcotic use by comparing the SSRF and NOM groups.

**(A)**										
		**Number of Patients**								
**First Author**	**DOI**	**Total**	**SSRF**	**NOM**	**MV** **MD (*p*)**	**Pneumonia** **RR (*p*)**	**ICU-LOS** **MD (*p*)**	**HLOS** **MD (*p*)**	**Mortality** **RR (*p*)**	**Tracheostomy RR (*p*)**
[3]	doi:10.1186/s13017-025-00581-y	1,078,795	N/A	N/A	−0.03 (0.81) *	1.06 (0.66) *	−037 (0.04)	1.92 (0.006)	0.53 (0.0001)	1.37 (0.07) *
[7]	doi:10.1016/j.injury.2020.07.009	4565	793	3772	−6.01 (0.39) *	0.66 (0.008)	−2.93 (0.32) *	−5.78 (0.01)	0.32 (0.001)	N/A
[8]	doi:10.1186/s13017-024-00540-z	862	423	439	−4.62 (0.003)	0.57 (0.02)	−3.05 (0.03)	3.79 (0.18) *	0.53 (0.19) *	0.70 (0.26) *
[9]	doi:10.1016/j.ijsu.2020.09.010	538	260	278	−4.93 (0.01)	0.40 (0.001)	−5.72 (0.001)	−8.48 (0.001)	0.75 (0.53) *	0.61 (0.18) *
[10]	doi:10.1007/s00068-021-01606-2	157	79	78	−1.36 (0.01)	0.50 (0.16) *	N/A	N/A	N/A	0.58 (0.02)
[11]	doi:10.1308/rcsann.2021.0148	286	139	147	−6.3 (0.05)	0.46 (0.001)	−6.46 (0.001)	−7.18 (0.1) *	0.54 (0.26) *	0.58 (0.26) *
[12]	doi:10.1016/j.jss.2022.02.055	10,892	2960	7932	−1.67 (0.03)	0.77 (0.0001)	−1.29 (0.03)	−0.36 (0.03)	0.63 (0.04)	0.63 (0.03)
[13]	doi:10.1016/j.injury.2024.111705	752	372	380	−4.49 (0.001)	0.58 (0.02)	−3.84 (0.001)	−2.36 (0.45) *	0.62 (0.55) *	0.71 (0.02)
[14]	doi:10.21037/jtd-23-1117	2440	1015	1425	−5.23 (0.02)	0.46 (0.04)	−4.0 (0.0008)	−6.54 (0.00001)	0.94 (0.90) *	0.67 (0.10) *
[15]	doi:10.1016/j.jamcollsurg.2020.10.022	18,018	N/A	N/A	−4.86 (N/A)	0.37 (N/A)	−5.96 (N/A)	−9.90 (N/A)	0.33 (N/A)	0.41 (N/A)
[16]	doi:10.1007/s00068-023-02339-0	544	269	275	−4.34 (0.01)	0.5 (0.01)	−4.62 (0.01)	−5.39 (0.08) *	0.56 (0.29) *	0.59 (0.06) *
**(B)**										
		**Number of Patients**								
**First Author**	**DOI**	**Total**	**SSRF**	**NOM**	**QoL/Pain**					
[3]	doi:10.1186/s13017-025-00581-y	1,078,795	N/A	N/A	No assessment was made of these parameters					
[7]	doi:10.1016/j.injury.2020.07.009	4565	793	3772	No assessment was made of these parameters					
[8]	doi:10.1186/s13017-024-00540-z	862	423	439	No assessment was made of these parameters					
[9]	doi:10.1016/j.ijsu.2020.09.010	538	260	278	Significantly shorter duration of narcotics in SSRF over NOM					
[10]	doi:10.1007/s00068-021-01606-2	157	79	78	Marked improvement in pain associated with cough and deep breath in SSRF over NOM					
[11]	doi:10.1308/rcsann.2021.0148	286	139	147	No statistical differences between the SSRF and NOM groups					
[12]	doi:10.1016/j.jss.2022.02.055	10,892	2960	7932	No statistical analysis was performed in the study					
[13]	doi:10.1016/j.injury.2024.111705	752	372	380	No statistical differences between the SSRF and NOM groups					
[14]	doi:10.21037/jtd-23-1117	2440	1015	1425	MD −2.24; *p* < 0.00001					
[15]	doi:10.1016/j.jamcollsurg.2020.10.022	18,018	N/A	N/A	No assessment was made of these parameters					
[16]	doi:10.1007/s00068-023-02339-0	544	269	275	No assessment was made of these parameters					

Abbreviations: SSRF: surgical stabilisation of rib fractures; NOM: non-operative management; MV: mechanical ventilation; MD: mean difference; RR: risk ratio; ICU-LOS: intensive care unit length of stay; HLOS: hospital length of stay; N/A: the data were not available from the original paper; *: the asterisk flag denotes the non-significant *p*-value. QoL: quality of life.

## Data Availability

No new data were created or analyzed in this study.

## Supplementary Materials

The following supporting information can be downloaded at: https://www.mdpi.com/article/10.3390/jcm15103648/s1, Table S1: AMSTAR 2 Full Assessment with Critical Domains and Overall Rating (11 Studies).

## Abbreviations

The following abbreviations are used in this manuscript:

ICU	Intensive care unit
SSRF	Surgical stabilization of rib fractures
NOM	Non-operative management
MeSH	Medical subject headings
MV	Mechanical ventilation
QoL	Quality of life
NPS	Numeric pain score
MD	Mean difference
RR	Relative ratio
ICU-LOS	Intensive care length of stay
HLOS	Hospital length of stay
N/A	Not available
CT	Computed tomography
TEA	Thoracic epidural anesthesia
PVB	Paravertebral block
ESPB	Erector spinae plane block
