# Supplementary material for: An Overview of Meta-Analyses on the Surgical Stabilization of Rib Fractures in Adults: A Narrative Umbrella Review (2020–2025)
Source: J Clin Med. 2026 May 9;15(10):3648. doi: 10.3390/jcm15103648 (PMC13206892; doi:10.3390/jcm15103648)
Supplement: Supplementary file 1 [file jcm-15-03648-s001.zip › jcm-4237185-supplementary.pdf]

Supplementary Table S1. AMSTAR 2 Full Assessment with Critical Domains and Overall Rating (11 Studies)

Hisamune 2024

Domain	Judgment	Critical Domain
Protocol registered (D2)	Yes	Critical
Comprehensive search (D4)	Yes	Critical
List of excluded studies (D7)	Partial	Critical
Risk of bias assessment (D9)	Yes	Critical
Meta-analysis methods (D11)	Yes	Critical
RoB in interpretation (D13)	Partial	Critical
Publication bias (D15)	Partial	Critical
Research question	Yes	Non-critical
Study selection duplicate	Yes	Non-critical
Data extraction duplicate	Yes	Non-critical
Description of studies	Yes	Non-critical
Funding sources	Partial	Non-critical
Heterogeneity discussion	Yes	Non-critical
Conflict of interest	Yes	Non-critical

Overall AMSTAR 2 Rating: Moderate

Long 2020

Domain	Judgment	Critical Domain
Protocol registered (D2)	Yes	Critical
Comprehensive search (D4)	Yes	Critical
List of excluded studies (D7)	Partial	Critical
Risk of bias assessment (D9)	Yes	Critical
Meta-analysis methods (D11)	Yes	Critical
RoB in interpretation (D13)	Partial	Critical
Publication bias (D15)	Partial	Critical
Research question	Yes	Non-critical
Study selection duplicate	Yes	Non-critical
Data extraction duplicate	Yes	Non-critical
Description of studies	Yes	Non-critical
Funding sources	Partial	Non-critical
Heterogeneity discussion	Yes	Non-critical
Conflict of interest	Yes	Non-critical

Overall AMSTAR 2 Rating: Moderate

Zhao 2025

Domain	Judgment	Critical Domain
Protocol registered (D2)	Yes	Critical
Comprehensive search (D4)	Yes	Critical
List of excluded studies (D7)	No	Critical
Risk of bias assessment (D9)	Yes	Critical
Meta-analysis methods (D11)	Yes	Critical
RoB in interpretation (D13)	No	Critical
Publication bias (D15)	Partial	Critical
Research question	Yes	Non-critical
Study selection duplicate	Yes	Non-critical
Data extraction duplicate	Yes	Non-critical
Description of studies	Yes	Non-critical
Funding sources	Partial	Non-critical
Heterogeneity discussion	Yes	Non-critical
Conflict of interest	Yes	Non-critical

Overall AMSTAR 2 Rating: Low

Apampa 2022

Domain	Judgment	Critical Domain
Protocol registered (D2)	No	Critical
Comprehensive search (D4)	Yes	Critical
List of excluded studies (D7)	No	Critical
Risk of bias assessment (D9)	Yes	Critical
Meta-analysis methods (D11)	Yes	Critical
RoB in interpretation (D13)	Partial	Critical
Publication bias (D15)	Partial	Critical
Research question	Yes	Non-critical
Study selection duplicate	Yes	Non-critical
Data extraction duplicate	Yes	Non-critical
Description of studies	Yes	Non-critical
Funding sources	Partial	Non-critical
Heterogeneity discussion	Yes	Non-critical
Conflict of interest	Yes	Non-critical

Overall AMSTAR 2 Rating: Moderate

Craxford 2022

Domain	Judgment	Critical Domain
Protocol registered (D2)	No	Critical
Comprehensive search (D4)	Yes	Critical
List of excluded studies (D7)	No	Critical
Risk of bias assessment (D9)	Yes	Critical
Meta-analysis methods (D11)	Yes	Critical
RoB in interpretation (D13)	Partial	Critical
Publication bias (D15)	Partial	Critical
Research question	Yes	Non-critical
Study selection duplicate	Yes	Non-critical
Data extraction duplicate	Yes	Non-critical
Description of studies	Yes	Non-critical
Funding sources	Partial	Non-critical
Heterogeneity discussion	Yes	Non-critical
Conflict of interest	Yes	Non-critical

Overall AMSTAR 2 Rating: Moderate

Sharma 2024

Domain	Judgment	Critical Domain
Protocol registered (D2)	Yes	Critical
Comprehensive search (D4)	Yes	Critical
List of excluded studies (D7)	No	Critical
Risk of bias assessment (D9)	Yes	Critical
Meta-analysis methods (D11)	Yes	Critical
RoB in interpretation (D13)	Partial	Critical
Publication bias (D15)	Partial	Critical
Research question	Yes	Non-critical
Study selection duplicate	Yes	Non-critical
Data extraction duplicate	Yes	Non-critical
Description of studies	Yes	Non-critical
Funding sources	Partial	Non-critical
Heterogeneity discussion	Yes	Non-critical
Conflict of interest	Yes	Non-critical

Overall AMSTAR 2 Rating: Moderate

He 2023

Domain	Judgment	Critical Domain
Protocol registered (D2)	No	Critical
Comprehensive search (D4)	Yes	Critical
List of excluded studies (D7)	No	Critical
Risk of bias assessment (D9)	Yes	Critical
Meta-analysis methods (D11)	Yes	Critical
RoB in interpretation (D13)	No	Critical
Publication bias (D15)	Partial	Critical
Research question	Yes	Non-critical
Study selection duplicate	Yes	Non-critical
Data extraction duplicate	Yes	Non-critical
Description of studies	Yes	Non-critical
Funding sources	Partial	Non-critical
Heterogeneity discussion	Yes	Non-critical
Conflict of interest	Yes	Non-critical

Overall AMSTAR 2 Rating: Low

Ferreira 2023

Domain	Judgment	Critical Domain
Protocol registered (D2)	Yes	Critical
Comprehensive search (D4)	Yes	Critical
List of excluded studies (D7)	No	Critical
Risk of bias assessment (D9)	Yes	Critical
Meta-analysis methods (D11)	Yes	Critical
RoB in interpretation (D13)	Partial	Critical
Publication bias (D15)	Partial	Critical
Research question	Yes	Non-critical
Study selection duplicate	Yes	Non-critical
Data extraction duplicate	Yes	Non-critical
Description of studies	Yes	Non-critical
Funding sources	Partial	Non-critical
Heterogeneity discussion	Yes	Non-critical
Conflict of interest	Yes	Non-critical

Overall AMSTAR 2 Rating: Moderate

Choi 2021

Domain	Judgment	Critical Domain
Protocol registered (D2)	No	Critical
Comprehensive search (D4)	Yes	Critical
List of excluded studies (D7)	No	Critical
Risk of bias assessment (D9)	Partial	Critical
Meta-analysis methods (D11)	Yes	Critical
RoB in interpretation (D13)	No	Critical
Publication bias (D15)	Partial	Critical
Research question	Yes	Non-critical
Study selection duplicate	Yes	Non-critical
Data extraction duplicate	Yes	Non-critical
Description of studies	Yes	Non-critical
Funding sources	Partial	Non-critical
Heterogeneity discussion	Yes	Non-critical
Conflict of interest	Yes	Non-critical

Overall AMSTAR 2 Rating: Low

Wijffels 2020

Domain	Judgment	Critical Domain
Protocol registered (D2)	No	Critical
Comprehensive search (D4)	Yes	Critical
List of excluded studies (D7)	No	Critical
Risk of bias assessment (D9)	Partial	Critical
Meta-analysis methods (D11)	Yes	Critical
RoB in interpretation (D13)	No	Critical
Publication bias (D15)	Partial	Critical
Research question	Yes	Non-critical
Study selection duplicate	Yes	Non-critical
Data extraction duplicate	Yes	Non-critical
Description of studies	Yes	Non-critical
Funding sources	Partial	Non-critical
Heterogeneity discussion	Yes	Non-critical
Conflict of interest	Yes	Non-critical

Overall AMSTAR 2 Rating: Low

Sawyer 2022

Domain	Judgment	Critical Domain
Protocol registered (D2)	No	Critical
Comprehensive search (D4)	Yes	Critical
List of excluded studies (D7)	No	Critical
Risk of bias assessment (D9)	Partial	Critical
Meta-analysis methods (D11)	Yes	Critical
RoB in interpretation (D13)	No	Critical
Publication bias (D15)	Partial	Critical
Research question	Yes	Non-critical
Study selection duplicate	Yes	Non-critical
Data extraction duplicate	Yes	Non-critical
Description of studies	Yes	Non-critical
Funding sources	Partial	Non-critical
Heterogeneity discussion	Yes	Non-critical
Conflict of interest	Yes	Non-critical

Overall AMSTAR 2 Rating: Low
